# Systematic study of the effects of lowering low-density lipoprotein-cholesterol on regression of coronary atherosclerotic plaques using intravascular ultrasound

**DOI:** 10.1186/1471-2261-14-60

**Published:** 2014-05-02

**Authors:** Wen-Qian Gao, Quan-Zhou Feng, Yu-Feng Li, Yuan-Xin Li, Ya Huang, Yan-Ming Chen, Bo Yang, Cai-Yi Lu

**Affiliations:** 1 The Department of Cardiology, Chinese PLA General Hospital, Beijing 100853, China; 2 The First Department of Geriatric Cardiology, Chinese PLA General Hospital, Beijing 100853, China; 3 Navy Wangshoulu Clinics, Xicui Road, Beijing 100036, China

**Keywords:** Low-density lipoprotein-cholesterol, Coronary atherosclerotic plaque, Intravascular ultrasound, Coronary artery disease

## Abstract

**Background:**

Conflicting results currently exist on the effects of LDL-C levels and statins therapy on coronary atherosclerotic plaque, and the target level of LDL-C resulting in the regression of the coronary atherosclerotic plaques has not been settled.

**Methods:**

PubMed, EMBASE, and Cochrane databases were searched from Jan. 2000 to Jan. 2014 for randomized controlled or blinded end-points trials assessing the effects of LDL-C lowering therapy on regression of coronary atherosclerotic plaque (CAP) in patients with coronary heart disease by intravascular ultrasound. Data concerning the study design, patient characteristics, and outcomes were extracted. The significance of plaques regression was assessed by computing standardized mean difference (SMD) of the volume of CAP between the baseline and follow-up. SMD were calculated using fixed or random effects models.

**Results:**

Twenty trials including 5910 patients with coronary heart disease were identified. Mean lowering LDL-C by 45.4% and to level 66.8 mg/dL in the group of patients with baseline mean LDL-C 123.7 mg/dL, mean lowering LDL-C by 48.8% and to level 60.6 mg/dL in the group of patients with baseline mean LDL-C 120 mg/dL, and mean lowering LDL-C by 40.4% and to level 77.8 mg/dL in the group of patients with baseline mean LDL-C 132.4 mg/dL could significantly reduce the volume of CAP at follow up (SMD −0.108 mm^3^, 95% CI −0.176 ~ −0.040, *p* = 0.002; SMD −0.156 mm^3^, 95% CI −0.235 ~ −0.078, *p* = 0.000; SMD −0.123 mm^3^, 95% CI −0.199 ~ −0.048, *p* = 0.001; respectively). LDL-C lowering by rosuvastatin (mean 33 mg daily) and atorvastatin (mean 60 mg daily) could significantly decrease the volumes of CAP at follow up (SMD −0.162 mm^3^, 95% CI: −0.234 ~ −0.081, *p* = 0.000; SMD −0.101, 95% CI: −0.184 ~ −0.019, *p* = 0.016; respectively). The mean duration of follow up was from 17 ~ 21 months.

**Conclusions:**

Intensive lowering LDL-C (rosuvastatin mean 33 mg daily and atorvastatin mean 60 mg daily) with >17 months of duration could lead to the regression of CAP, LDL-C level should be reduced by >40% or to a target level <78 mg/dL for regressing CAP.

## Background

It is universally accepted that high serum concentrations of low-density lipoprotein cholesterol (LDL-C) can lead to atherosclerosis and accelerate the progression of atherosclerosis which is main causes of coronary artery disease [1]. Disruption of coronary atherosclerotic plaque (CAP) with subsequent thrombus formation may lead to sudden cardiac death, acute myocardial infarction, or unstable angina [2]. The evidence showed that reducing LDL-C can prevent coronary heart disease (CHD) and improve survival of CHD based on results from multiple randomized controlled trials (RCTs) [3,4].

For many years coronary angiography (CAG) has been the gold standard method for the investigation of the anatomy of coronary arteries and measure the efficacy of anti-atherosclerotic drug therapies [5,6]. But changes in CAG are measured only in the vascular lumen and not in the vessel wall [7], where the atherosclerotic process is located. Intravascular ultrasound (IVUS) is superior to angiography in the detection of early plaque formation and changes in plaque volume [8-10]. Through IVUS, Takagi et al. found that pravastatin lowered serum cholesterol levels and reduced the progression of CAP in patients with elevated serum cholesterol levels in 1997 [11]. Since then, multiple RCTs and no RCT about the effect of lowering LDL-C therapy on the regression of coronary atherosclerosis have been performed [12-16]. But the results varied with the RCTs: intensive LDL-C lowering therapy could reduce the progression of the plaques [12]; the mild LDL-C lowering did not [14-16]. The meta-analysis by Bedi et al. [17] evaluated the effects of LDL-C lowering on CAP by comparing statins with control therapy, and demonstrated that treatment with statins could slow atherosclerotic plaque progression and lead to plaque regression. The meta-analysis by Tian et al. [18] showed that CAP could be regressed in group of patients with <100 mg of LDL-C level at follow up. But so far, there are no systematic reviews of the effects of LDL-C levels on CAP, and the targets of LDL-C level that could result in the regression of the plaques have not been settled.

In this study, we conducted meta-analyses to summarize findings from the current trials on LDL-C lowering therapy retarding the progression of the CAP and to identify the targets of LDL-C resulting in the regression of the CAP for guiding the LDL-C lowering therapy. Effect of different statins on the progression of the CAP was also investigated.

## Methods

### Search strategy and selection criteria

An electronic literature search was performed to identify all relevant studies published in PubMed, EMBASE, and Cochrane databases in the English language from Jan. 1, 2000 to Jan. 1, 2014, using the terms “atherosclerosis” and “cholesterol blood level”. The references of the studies were also searched for relevant studies. Studies were included using the following criteria: 1) randomized controlled or prospective, blinded end-points trials in which patients with CHD were assigned to LDL-C lowering therapy or placebo, and its primary end point was CAP change detected by IVUS; 2) report of LDL-C levels at baseline and follow-up (in each arm) or the level of LDL-C which can be calculated from the data in the paper (as in the trial by Yokoyama M [15], in which the LDL-C concentrations in control arm were directly extracted from the figure); 3) data on the volume of CAP, detected in IVUS at baseline and follow-up (in each arm), and volume of CAP was calculated as vessel volume minus lumen volume; Exclusion criteria were: 1) only CAP area or volume index or percent atheroma volume were detected by IVUS; 2) the levels of LDL-C at baseline or follow-up were not provided; and 3) target plaques were unstable.

### Data extraction and quality assessment

Two investigators independently reviewed all potentially eligible studies and collected data on patient and study characteristics (author, year, design, sample size, the measures of LDL-C lowering, LDL-C levels, follow-up duration, and plaque volume), and any disagreement was resolved by consensus. The primary end point of this study was progression or regression of CAP detected by IVUS. Quality assessments were evaluated with Jadad quality scale [19].

### Data synthesis and analysis

Continuous variables (change of CAP volume from baseline to follow-up) were analyzed using standardized mean differences (SMD).

The trials may have control arm and multiple active treatment arms, changes of plaque volume in every arms were used for pooled analysis. According to the levels and the reducing percentage of LDL-C at follow-up, the arms were grouped to following groups: ≤70, >70 ≤ 100HP (>70 ≤ 100 mg and reducing percentage ≥30%), >70 ≤ 100MP (>70 ≤ 100 mg and reducing percentage ≥0 < 30%), >70 ≤ 100LP (>70 ≤ 100 mg and reducing percentage <0%), >100 mg/dL; and <0, ≥0 < 30, ≥30 < 40, ≥40 < 50, ≥50% respectively, to investigate the effect of different levels of LDL-C at follow up on CAPs. According to different statins, the arms were grouped to following groups: rosuvastatin, atorvastatin , pitavastatin, simvastatin, fluvastatin and pravastatin group, to investigate the effect of different statins on CAPs. The volume of CAP at follow up was compared with that at baseline to evaluate effect of LDL-C levels on regression of CAP.

Heterogeneity across trials (arms) was assessed via a standard χ^*2*^ test with significance being set at *p* < 0.10 and also assessed by means of *I*^*2*^ statistic with significance being set at *I*^*2*^ > 50%. Pooled analyses were calculated using fixed-effect models, whereas random-effect models were applied in case of significant heterogeneity across studies (arms). Sensitivity analyses (exclusion of one study at one time) were performed to determine the stability of the overall effects of LDL-C levels. Additionally, publication bias was assessed using the Egger regression asymmetry test. Mean LDL-C level and follow up duration of groups were calculated by descriptive statistics. A two-sided *p* values < 0.05 was considered statistically significant. Statistical analyses were performed using STATA software 12.0 (StataCorp, College Station, Texas) and Review Manager V5.2 (Copenhagen: The Nordic Cochrane Centre, The Cochrane Collaboration, 2012).

## Results

### Eligible studies

**Figure 1:**
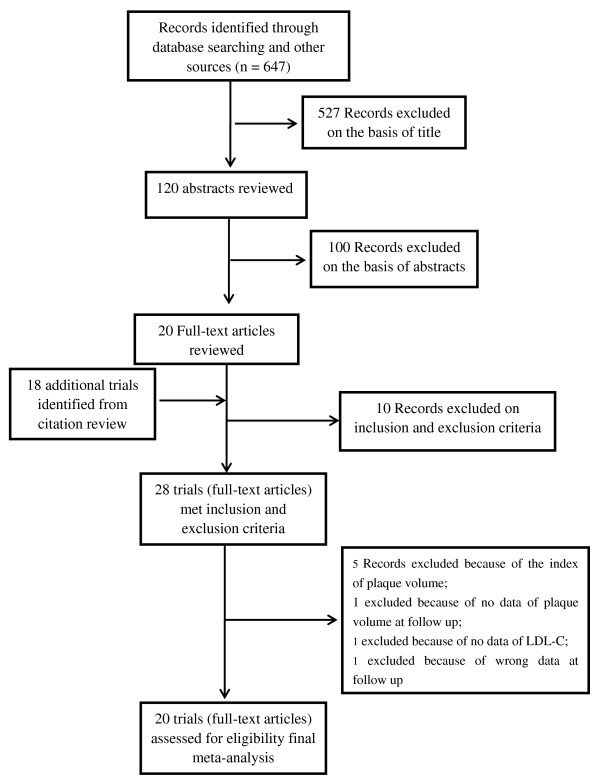
Flow diagram of study-screening process.

The flow of selecting studies for the meta-analysis is shown in Figure 1. Briefly, of the initial 647 articles, one hundred and twenty of abstracts were reviewed, resulting in exclusion of 100 articles, and 20 articles were reviewed in full text, resulting in exclusion of 10 trials and inclusion of 18 additional trials. Twenty two RCTs [12-16,20-31], [32-36] and six blinded end-points trial [37-42] were carefully evaluated. Five trials were excluded because of specific the index of plaque (volume index in TRUTH [24], trial by Kovarnik T [31], by Hattori K [42], and by Petronio AS [32]; area in LACMART [38]); GAIN [20] excluded because of no data of plaque volume at follow up; trial by Zhang X [25] excluded because of no data of LDL-C; trial by Hong YJ [30] excluded because of wrong data at follow up. Sixteen RCT (ESTABLISH [14], REVERSAL [13], A-PLUS [21], ACTIVATE [22], ILLUSTRATE [23], JAPAN-ACS [12], REACH [26], SATURN [28], ARTMAP [29], ERASE [34], STRADIVARIUS [35], PERISCOPE [36], and trials by Yokoyama M [15], by Kawasaki M [16], by Hong MK [27], and Tani S [33]) and four blinded end-points trial (ASTEROID [37], COSMOS [40], trial by Jensen LO [39] and trial by Nasu K [41]) were finally analyzed.

The characteristics of the included trials were shown in Table 1. Among the 20 trials, there were 15 trials assessing statins (statin vs. usual care in 6 trials [14-16,26,33,41]; intensive statin vs. moderate statin treatment in 5 trials [12,13,27-29]; follow up vs baseline in 3 trial [37,39,40], before acute coronary syndrome (ACS) vs after ACS in one trial [34]), 2 trials assessing enzyme acyl–coenzyme A: cholesterol acyltransferase (ACAT) inhibition (vs. placebo, both on the basis of mean LDL-C < 102 after background lipid-lowering therapy with statins in 62-79% of patients) [21,22], one trial assessing cholesteryl ester transfer protein (CETP) inhibitor torcetrapib (vs. statins on the basis of LDL-C ≤ 100 by statins) [23], one trial assessing a decreasing obesity drug: rimonabant (vs. placebo, on the basis of statins therapy) [35], and one trial assessing glucose-lowering agents (pioglitazone vs glimepiride on the basis of statins therapy) [36]. In three trials [12,14,34] with acute coronary syndrome, all target plaques were selected in non-culprit vessels. Overall, 5910 patients with CHD underwent serial IVUS examination for evaluating regression of CAP. Follow-up periods ranged from 2 to 24 months. The levels of LDL-C of each arm at baseline and follow-up were shown in Table 2.

**Table 1:** Features of participating trials

**Authors and trial name**	**Trial type and location**	**Objective**	**Year**	**N T/C**	**Study population**	**LDL-C at follow up**	**LDL-C reducing percentage**	**Treatments**	**Follow up**	**Main Results or Conclusion**
Okazaki S [14];ESTABLISH	RCT: prospective, open-label, randomized, single center study. Japan	Effects of statins on changes in plaque by IVUS	2004	24/24	ACS	70/119	-44/-0.004	Ato 20 vs Diet	6	Plaque volume was sigificantly reduced in the Ato group compared with the control group.
Nissen SE [13]; REVERSAL	RCT: Double-blind, randomized active control multicenter trial; USA	Effects of statins (intensive or moderate) on changes in plaque by IVUS	2004	253/249	CAD	79/110	-46/-25	Ato 80 vs Pra40	18	Ato reduced progression of coronary plaque compared with Pra. Compared with baseline values, Ato had no change in atheroma burden, whereas patients treated with Pra showed progression of coronary plaque.
Tardif JC [21]; A-PLUS	RCT: international, multicenter, double-blind, placebo-controlled, randomized trial. Canada, USA	Effects of different dosage of avasimibe on changes in plaque by IVUS	2004	108/98/ 117/109	CAD	100/102/ 101/91	7.8/9.1/ 10.9/1.7	Ava50, 250, and 750 vs Placebo on the basis of LDL-C<125	18	Avasimibe did not favorably alter coronary atherosclerosis as assessed by IVUS.
Jensen LO [39]	Open non placebo controlled serial investigation; blinded end-points. Denmark	To investigate the effect of lipid lowering by simvastatin on coronary atherosclerotic plaque volumes and lumen.	2004	40	CAD	85	-46.3	Sim 40	15	Lipid-lowering therapy with Sim is associated with a significant plaque regression in coronary arteries.
Yokoyama M [15]	RCT: randomized, single center. Japan	Effects of statins on changes in plaque by IVUS	2005	29/30	Stable angina	87/124	-35/-0.075	Ato 10 vs Diet	6	Treatment with Ato may reduce volumes of coronary plaques.
Kawasaki M [16]	RCT: randomization, open-label, single-center study. Japan	Effects of statins on changes in plaque by IVUS	2005	17/18/17	Stable angina	95/102/149	-39/-32/-0.02	Ato 20, Pra 20 vs Diet	6	Treatment with Ato and Pra may not significantly reduce volumes of coronary plaques.
Tani S [33]	RCT: a prospective, single-center, randomized, open trial. Japan	Investigated the effects of pravastatin on the serum levels of MDA-LDL and coronary atherosclerosis.	2005	52/23	Stable angina	104/120	-20/-2.4	Pra 10-20 vs con	6	Plaque volume was sigificantly reduced in the Pra group compared with the control group.
Nissen SE [22]; ACTIVATE	RCT: randomized, multicenter. USA	Effects of pactimibe on changes in plaque by IVUS	2006	206/202	CAD	91/86	-9.6/-14.9	Pac100 vs Placebo	18	Pac is not an effective strategy for limiting atherosclerosis and may promote atherogenesis.
Nissen SE [37]; ASTEROID	Prospective, open-label blinded end-points. USA, Germany, France, Canada	Effects of Statins with different levels of LDL-C on changes in plaque by IVUS	2006	349	CAD	61	-53.2	Ros 40	24	Therapy using Ros can result in significant regression of atherosclerosis.
Yamada T [26]; REACH	RCT: open-labeled, randomized, multicenter study. Japan	Evaluate the effect of marked reduction of LDL-C in patients with CHD on progression of atherosclerosis.	2007	26/32	Stable angina	83/115	-43/0	Ato 5 vs Con	12	Ato treatment prevented the further progression of atherosclerosis by maintaining LDL-C below 100 mg/dl in patients with CHD.
Nissen SE [23]; ILLUSTRATE	RCT: prospective, randomized, multicenter, double-blind clinical trial. North America or Europe	Effects of CETP inhibitor on changes in plaque by IVUS	2007	446/464	CAD	87/70	6.6/-13.3	Ato10-80 vs Ato+Tor 60 on the basis of LDL-C≤100 by Ato	24	The Tor was associated with a substantial increase in HDL-C and decrease in LDL–C, and there was no significant decrease in the progression of coronary atherosclerosis.
Nissen SE [36]; PERISCOPE	RCT: prospective, randomized, multicenter, double-blind clinical trial. USA	To compare the effects of pioglitazone, and glimepiride on the progression of coronary atherosclerosis in patients with type 2 diabete and CAD	2008	181/179	CAD, DM	96.1/95.6	1.8/2.2	Gli1-4 mg vs Pio 15-45 mg on bases of statins therapy	18	In patients with type 2 diabetes and CAD, treatment with Pio resulted in a significantly lower rate of progression of coronary atherosclerosis compared with Gli.
Nissen SE [35]; STRADIVARIUS	RCT: Randomized, double-blinded, placebo-controlled, 2-group, parallel-group trial. North America, Europe, and Australia	The effect of rimonabant on regression of coronary disease in patients with the metabolic syndrome and CAD	2008	335/341	CAD,Obesity	87.6/86.3	-4.7/-3.6	Rim 20 mg vs Placebo on bases of statins therapy	18	Rim can reduce progression of coronary plaque, and increase HDL-C levels, decrease triglyceride levels.
Hiro T [12]; JAPAN-ACS	RCT: prospective, randomized, open-label, parallel group, multicenter. Japan	Effects of statins on changes in plaque by IVUS	2009	127/125	ACS	84/81	-36/-36	Ato 20 vs Pit 4	10	The administration of Pit or Ato in patients with ACS equivalently resulted in significant regression of coronary plaque volume.
Takayama T; COSMOS [40]	Prospective, open-label blinded end-points multicenter trial. Japan	Evaluate the effect of rosuvastatin on plaque volume in patients with stable CAD, including those receiving prior lipid-lowering therapy	2009	126	Stable angina	83	-38.6	Ros <20	14	Ros exerted significant regression of coronary plaque volume in Japanese patients with stable CAD.
Rodés-Cabau; ERASE [34]	RCT: multicenter randomized placebo-controlled. Canada	Evaluate the early effects of newly initiated statin therapy on coronary atherosclerosis as evaluated by IVUS.	2009	38/36	ACS	77/63	8.5/-37	Before ACS vs After ACS	<2	Newly initiated statin therapy is associated with rapid regression of coronary atherosclerosis.
Nasu K [41]	Prospective and multicenter study with nonrandomized and non-blinded design, but blinded end. Japan	Evaluate the effect of treatment with statins on the progression of coronary atherosclerotic plaques of a nonculprit vessel by serial IVUS.	2009	40/39	Stable angina	98.1/121	-32.3/-1.1	Flu 60 vs Con	12	One-year lipid-lowering therapy by Flu showed significant regression of plaque volume.
Hong MK [27]	RCT: randomized control trial. Korea.	Evaluated the effects of statin treatments for each component of coronary plaques.	2009	50/50	Stable angina	78/64	-34.5/-44.8	Sim 20 vs Ros 10	12	Statin treatments might be associated with significant changes in necrotic core and fibrofatty plaque volume.
Nicholls SJ; SATURN [28]	RCT: a prospective, randomized, multicenter, double-blind clinical trial. USA	Compare the effect of these two intensive statin regimens on the progression of coronary atherosclerosis.	2011	519/520	CHD	70.2/62.6	-41.5/-47.8	Ato 80 vs Ros 40	24	Maximal doses of Ros and Ato resulted in significant regression of coronary atherosclerosis.
Lee CW [29]; ARTMAP	RCT: a prospective, single-center, open-label, randomized comparison trial. Korea.	Compared the effects of atorvastatin 20 mg/day versus rosuvastatin 10 mg/day on mild coronary atherosclerotic plaques.	2012	143/128	Stable angina	56/53	-47/-49	Ato 20 vs Ros 10	6	Usual doses of Ato and Ros induced significant regression of coronary atherosclerosis in statin-naive patients.

Abbreviations: RCT, randomized controlled trials; T, treatment group; C, control group IVUS, Intravascular ultrasound; CAD, Coronary artery disease; ACS, Acute coronary syndrome; CHD, Coronary heart disease; Ato, Atorvastatin; Ros, Rosuvastatin; Pra, Pravastatin; Pit, Pitavastatin; Sim, Simvastatin; Flu, Fluvastatin; Con, Control; Pac, Pactimibe; Tor, Torcetrapib, Ava 50, 250, 750, Avasimibe 50, 250, 750 mg; T/C, Treat/Control; Gli, Glimepiride; Pio, Pioglitazone; Rim, Rimonabant.

**Table 2:** The levels of LDL-C at baseline and follow up in each arm of included trials

**Authors**	**Trial name**	**Management in each arm**	**N**	**LDL-C level**	
				**At Baseline**	**At Follow-up**
Tardif JC	A-PLUS	Avasimibe50	108	92.8 ± 1.7	100*
Tardif JC	A-PLUS	Avasimibe250	98	93.4 ± 1.6	101.9*
Tardif JC	A-PLUS	Avasimibe750	117	91.4 ± 1.6	101.4*
Tardif JC	A-PLUS	Placebo	109	89.6 ± 1.6	91.1*
Okazaki S	ESTABLISH	Control	24	123.9 ± 35.3	119.4 ± 24.6
Okazaki S	ESTABLISH	Atorvastatin	24	124.6 ± 34.5	70.0 ± 25.0
Yokoyama M		Control	30	131.5 ± 23#	124.5 ± 24.1#
Yokoyama M		Atorvastatin	29	133 ± 13	87 ± 29
Nissen SE	REVERSAL	Atorvastatin	253	150.2 ± 27.9	78.9 ± 30.2
Nissen SE	REVERSAL	Pravastatin	249	150.2 ± 25.9	110.4 ± 25.8
Nissen SE	ACTIVATE	Pactimibe	206	101.4 ± 27.7	91.3
Nissen SE	ACTIVATE	Placebo	202	101.5 ± 31.1	86.4
Nissen SE	ILLUSTRATE	Atorvastatin	446	84.3 ± 18.9	87.2 ± 22.6
Nissen SE	ILLUSTRATE	Atorva+torcetrapib	464	83.1 ± 19.7	70.1 ± 25.4
Kawasaki M		Control	17	152 ± 20	149 ± 24
Kawasaki M		Pravastatin	18	149 ± 19	102 ± 13
Kawasaki M		Atorvastatin	17	155 ± 22	95 ± 15
Hiro T	JAPAN-ACS	Pitavastatin	125	130.9 ± 33.3	81.1 ± 23.4
Hiro T	JAPAN-ACS	Atorvastatin	127	133.8 ± 31.4	84.1 ± 27.4
Nissen SE	ASTEROID	Rosuvastatin	349	130.4 ± 34.3	60.8 ± 20.0
Takayama T	COSMOS	Rosuvastatin	126	140.2±31.5	82.9±18.7
Lee CW	ARTMAP	Atorvastatin	143	110 ± 31	56 ± 18
Lee CW	ARTMAP	Rosuvastatin	128	109 ± 31	53±18
Yamada T	REACH	Atorvastatin	26	123 ± 17	83 ± 22
Yamada T	REACH	Control	32	115 ± 14	115 ± 30
Nasu K		Fluvastatin	40	144.9 ± 31.5	98.1 ± 12.7
Nasu K		Control	39	122.3 ± 18.9	121.0 ± 21.2
Nicholls SJ	SATURN	Atorvastatin	519	119.9 ± 28.9	70.2 ± 1.0
Nicholls SJ	SATURN	Rosuvastatin	520	120.0 ± 27.3	62.6 ± 1.0
Hong MK		Simvastatin	50	119 ± 30	78 ± 20
Hong MK		Rosuvastatin	50	116 ± 28	64 ± 21
Tani S		Pravastatin	52	130 ± 38	104 ± 20
Tani S		Control	23	123 ± 28	120 ± 30
Rodés-C Bef	ERASE	Statins before ACS	38	71 ± 23	77 ± 25
Rodés-C Aft	ERASE	Statins after ACS	36	100 ± 30	63 ± 17
Jensen LO		Simvastatin	40	158.7 ± 30.6	85.1 ± 22.1
Nissen SE	PERISCOPE	Statins+Gli	181	94.4 ± 32.9	96.1 ± 30.4
Nissen SE	PERISCOPE	Statins+Pio	179	93.5 ± 30.7	95.6 ± 28.9
Nissen SE	STRADIVARIUS	Statins+Rim	335	91.9 ± 27.9	87.6 ± 30.5
Nissen SE	STRADIVARIUS	Statins+Con	341	89.5 ± 32.2	86.3 ± 30.3

Note: *calculated on the bases of baseline levels and change percentage at follow up [21].

# calculated according to Figure 2 in the paper [15].

**Figure 2:**
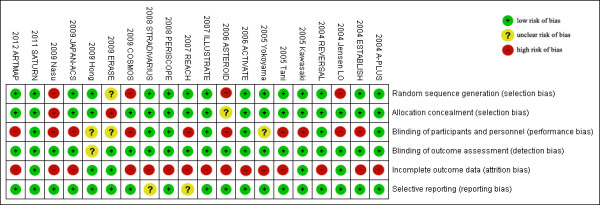
Methodological quality summary of each included trial.

**Figure 3:**
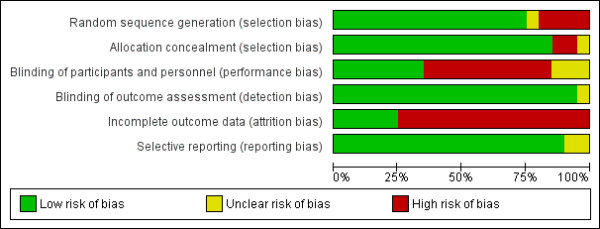
Methodological quality graph: each methodological quality item presented as percentages across all included studies.

Risk of bias of included studies, evaluated through Cochrane’s methods, showed an overall acceptable quality of selected trials (Figures 2 and 3).

### The effect of the levels of LDL-C at follow-up on regression of coronary atherosclerotic plaque

**Figure 4:**
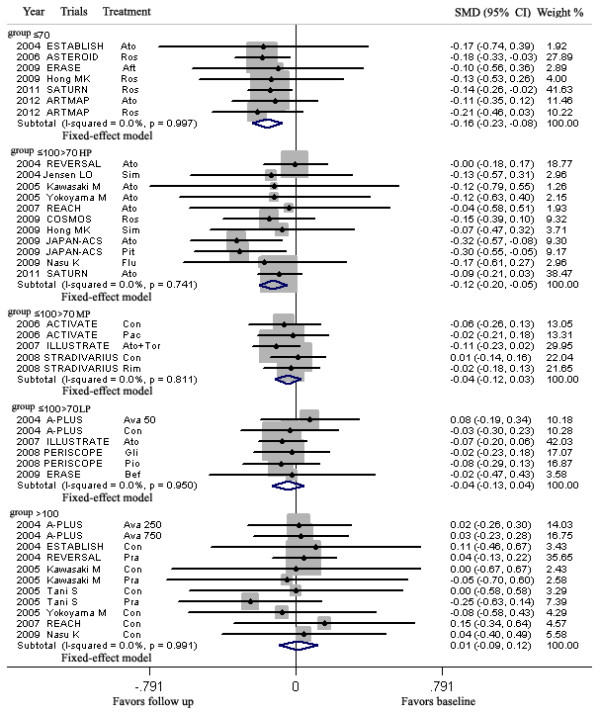
**Meta-analysis of the effects of reduction levels of LDL-C at follow up on the regression of coronary atherosclerotic plaque.** Abbreviations: Ato, Atorvastatin; Ros, Rosuvastatin; Pra, Pravastatin; Pit, Pitavastatin; Sim, Simvastatin; Flu, Fluvastatin; Con, Control; Pac, Pactimibe; Tor, Torcetrapib, Ava 50, 250, 750, Avasimibe 50, 250, 750 mg; Bef, before ACS; Aft, after ACS; Gli, Glimepiride; Pio, Pioglitazone; Rim, Rimonabant.

LDL-C lowering in group ≤70 and >70 ≤ 100HP mg/dL could lead to regression of CAP, but LDL-C lowering in group >70 ≤ 100MP, >70 ≤ 100LP and >100 mg/dL could not (Figure 4, Table 3).

**Table 3:** Results of meta-analysis in each group and mean CAP volume in each group at baseline and follow up

**Group**	**Included arms (case)**	**CAP Volume at Baseline (mm**^**3**^**)**	**CAP Volume at Follow up (mm**^**3**^**)**	**Pooled SMD (95% CI, *p *)**	**Heterogeneity test**		**Sensitivity analyses**		**Egger’s test**
					**χ**^***2***^**test ( *p *)**	***I***^***2***^	**Lower SMD (95% CI)**	**Upper SMD (95% CI)**	
≤70 mg	7 (1250)	177.1±41.9	125.9±38.6	-0.156 (-0.235~ -0.078, 0.000)	0.57 (0.997)	0	-0.146 (-0.238~ -0.054)	-0.167 (-0.270~ -0.064)	0.835
							Without 2006 ASTEROID Ros	Without 2011 SATURN Ros	
>70≤100HP mg	11 (1352)	129.7±72.3	123.8±69.8	-0.123 (-0.199~ -0.048, 0.001)	6.83 (0.741)	0	-0.103 (-0.182~ -0.024)	-0.151 (-0.235~ -0.067)	0.501
							Without 2009 JAPAN-ACS Ato	Without 2004 REVERSAL Ato	
>70≤100MP mg	5 (1548)	195.8±2.3	191.8±4.7	-0.045 (-0.115~ -0.026, 0.215)	1.59 (0.811)	0	-0.016 (-0.103~ -0.066)	-0.061 (-0.140~ -0.019)	0.500
							Without 2007 ILLUSTRATE Ato+Tor	Without 2008 STRADIVARIUS Con	
>70≤100LP mg	6 (1061)	201.2±15.1	197.3±15.0	-0.045 (-0.130~0.040, 0.301)	1.14 (0.950)	0	-0.024 (-0.136~ 0.087)	-0.059 (-0.148~ 0.031)	0.241
							Without 2007 ILLUSTRATE Ato	Without 2004 A-PLUS Ava 50	
>100 mg	10 (669)	175.9±86.4	178.7±89.1	0.017 (-0.090~0.124, 0.757)	2.37 (0.984)	0	-0.000 (-0.135~ 0.136)	0.039 (-0.073~ 0.151)	0.692
							Without 2004 REVERSAL Pro	Without 2005 Tani S Pra	
<0%	8 (1276)	201.2±13.8	198.3±13.8	-0.034 (-0.111~ 0.044, 0.396)	1.55 (0.981)	0	-0.012 (-0.109~ 0.084)	-0.044 (-0.125~ 0.037)	0.087
							Without 2007 ILLUSTRATE Ato	Without 2004 A-PLUS Ava 50	
>0≤30%	13 (2014)	188.6±51.7	186.3±52.7	-0.032 (-0.093~ 0.030, 0.315)	4.59 (0.970)	0	-0.010 (-0.080~ 0.061)	-0.042 (-0.108~ 0.024)	0.537
							Without 2007 ILLUSTRATE Ato+Tor	Without 2004 REVERSAL Pra	
>30≤40%	10 (594)	102.9±96.9	94.3±90.4	-0.199 (-0.314~ -0.085, 0.001)	3.10 (0.960)	0	-0.166 (-0.295~ -0.038)	-0.214 (-0.342~ -0.085)	0.024
							Without 2009 JAPAN-ACS Ato	Without 2009 COSMOS Ros	
>40≤50%	8 (1677)	157.8±37.8	150.7±36.3	-0.108 (-0.176~ -0.040, 0.002)	2.50 (0.927)	0	-0.093 (-0.174~ -0.011)	-0.126 (-0.200~ -0.053)	0.605
							Without 2011 SATURN Ros	Without 2004 REVERSAL Ato	
>50%	1 (349)	212.2±81.3	197.5±79.1	-0.183 (-0.332~ -0.035, 0.016)					

In group ≤70 mg/dL (including seven arms) with mean 18.6 months of follow up and group >70 ≤ 100HP mg/dL (including eleven arms) with mean 17.4 months of follow up, the volumes of CAP (125.9, 123.8 mm^3^ respectively) at follow up were significantly decreased, compared with the volumes (177.1, 129.7 mm^3^ respectively) at baseline [SMD −0.156 mm^3^, 95% CI (confidence interval) -0.235 ~ −0.078, *p* = 0.000; SMD −0.123 mm^3^, 95% CI −0.199 ~ −0.048, *p* = 0.001; respectively]. There was no significant heterogeneity among arms (χ^*2*^ for heterogeneity = 0.57, *p* =0.997, *I*^2^ = 0% for group ≤70 mg/dL; χ^*2*^ for heterogeneity = 6.83, *p* =0.741, *I*^2^ = 0% for group >70 ≤ 100HP mg/dL).

Sensitivity analyses suggested that LDL-C lowering in group ≤70 and >70 ≤ 100HP mg/dL could lead to regression of CAP with reduction of the CAP volume ranged from −0.146 mm^3^ (SMD, 95% CI: −0.238 ~ −0.054) when the arm of 2006 ASTEROID Ros was omitted to −0.167 mm^3^ (SMD, 95% CI: −0.270 ~ −0.064) when the arm of 2011 SATURN Ros was omitted; and from −0.103 mm^3^ (SMD, 95% CI: −0.182 ~ −0.024) when the arm of 2009 JAPAN-ACS Ato was omitted to −0.151 mm^3^ (SMD, 95% CI: −0.235 ~ −0.067) when the arm of 2004 REVERSAL Ato was omitted. No publication bias was found, the values of *p* by Egger’s test for group ≤70 and >70 ≤ 100HP mg/dL were 0.835, 0.501 respectively.

In group >100 mg/dL (including eleven arms) with mean 14.6 months of follow up, the volume of CAP at follow up was not significantly increased, compared with the volumes at baseline (SMD 0.013 mm^3^, 95% CI −0.092 ~ 0.118, *p* = 0.809). There was no significant heterogeneity among arms (χ^*2*^ for heterogeneity = 2.49, *p* =0.991, *I*^2^ = 0%).

Sensitivity analyses suggested that LDL-C lowering to >100 mg/dL at follow-up could still not lead to regression of CAP with reduction of the plaque volume ranged from −0.005 mm^3^ (95% CI −0.136 ~ 0.126) when the arm of 2004 REVERSAL Pro was omitted to 0.034 mm^3^ (SMD, 95% CI −0.075 ~ 0.143) when 2005 Tani S Pra was omitted. No publication bias was observed from the values of *p* (0.566) by Egger’s test.

Mean levels of LDL-C at baseline and follow up and mean reducing percentage of LDL-C in group ≤70, >70 ≤ 100HP, >70 ≤ 100MP, >70 ≤ 100LP and >100 mg/dL were showed in Table 4.

**Table 4:** Levels and reducing percentage of LDL-C and duration in each group

**Group**	**N**	**Mean LDL-C at Baseline (mg)**	**Mean LDL-C at Follow up (mg)**	**Mean Reducing percentage**	**Actual range of reducing percentage**	**Duration (month)**
≤70 mg	1250	120.0±8.2	60.6±3.5	48.8±3.3	37~53.2	18.6±8.2
>70≤100HP mg	1352	132.4±12.9	77.8±7.0	40.4±4.0	32.3~46.7	17.4±5.9
>70≤100MP mg	1548	91.3±6.9	82.4±8.2	9.1±4.5	3.6~14.9	19.8±2.7
>70≤100LP mg	1061	88.5±5.5	91.5±5.4	-4.7±2.5	-1.7~-8.5	19.9±4.5
>100 mg	699	125.1±24.4	110.0±9.3	8.3±15.6	-10.9~32	14.6±5.1
<0%	1276	89.1±5.3	93.2±6.2	-5.6±3.1	-1.7~-10.9	19.6±4.2
>0≤30%	2014	102.4±22.1	89.7±15.7	10.6±7.3	0~25	18.3±4.5
>30≤40%	594	132.6±11.4	83.3±7.7	36.1±1.9	32~39	10.3±3.1
>40≤50%	1677	123.7±13.4	66.8±8.0	45.4±2.8	41.5~49	19.4±6.9
>50%	349	130.4±34.3	60.8±20.0	53.2	53.2	24

### The effect of the LDL-C reducing percentage at follow-up on regression of CAP

**Figure 5:**
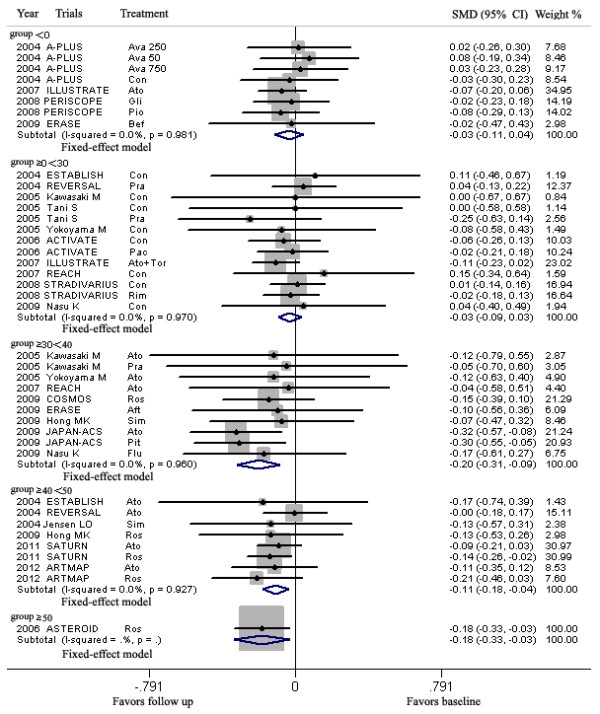
**Meta-analysis of the effects of reduction percentages of LDL-C at follow up on the regression of coronary atherosclerotic plaque.** Abbreviations: Ato, Atorvastatin; Ros, Rosuvastatin; Pra, Pravastatin; Pit, Pitavastatin; Sim, Simvastatin; Flu, Fluvastatin; Con, Control; Pac, Pactimibe; Tor, Torcetrapib, Ava 50, 250, 750, Avasimibe 50, 250, 750 mg; Bef, before ACS; Aft, after ACS; Gli, Glimepiride; Pio, Pioglitazone; Rim, Rimonabant.

LDL-C lowering in group ≥30 < 40, ≥40 < 50, ≥50% could lead to regression of CAP, but LDL-C lowering in group <0 and ≥0 < 30% could not (Figure 5, Table 3).

In group ≥30 < 40% (including ten arms) with mean 10.3 months of follow up, and group ≥40 < 50% (including eight arms) with mean 19.4 months of follow up, the volumes of CAP (94.3, 150.7 mm^3^ respectively) at follow up were significantly decreased, compared with the volumes (102.9, 157.8 mm^3^ respectively) at baseline (SMD −0.199 mm^3^, 95% CI −0.314 ~ −0.085, *p* = 0.001; SMD −0.108 mm^3^, 95% CI −0.176 ~ −0.040, *p* = 0.002; respectively). There was no significant heterogeneity among arms (χ^*2*^ for heterogeneity = 3.10, *P* = 0.960, *I*^2^ = 0%; χ^*2*^ for heterogeneity = 2.50, *p* =0.927, *I*^2^ = 0%; for group ≥30 < 40, and group ≥40 < 50 respectively).

Sensitivity analyses showed that LDL-C lowering in group ≥30 < 40% and group ≥40 < 50 could still lead to regression of CAP with reduction of the plaque volume ranged from −0.166 mm^3^ (95% CI −0.295 ~ −0.038) when the arm of 2009 JAPAN-ACS Ato was omitted to −0.214 mm^3^ (SMD, 95% CI −0.342 ~ −0.085) when 2009 COSMOS Ros was omitted; from −0.093 mm^3^ (95% CI −0.174 ~ −0.011) when the arm of 2011 SATURN Ros was omitted to −0.126 mm^3^ (SMD, 95% CI −0.200 ~ −0.053) when 2004 REVERSAL Ato was omitted respectively. Publication bias analysis suggested the values of *p* by Egger’s test were 0.024, 0.605 for group ≥30 < 40, and group ≥40 < 50 respectively.

In group <0 with mean 19.6 months of follow up and group ≥0 < 30% with mean 18.3 months of follow up, the volume of CAP at follow up was not significantly decreased, compared with the volumes at baseline (SMD −0.034 mm^3^, 95% CI −0.111 ~ 0.044, *p* = 0.396; SMD −0.032 mm^3^, 95% CI −0.093 ~ 0.030, *p* = 0.315 respectively). There was no significant heterogeneity among arms (χ^*2*^ for heterogeneity = 1.55, *p* =0.981, *I*^2^ = 0% for group <0%; χ^*2*^ for heterogeneity = 4.59, *p* =0.970, *I*^2^ = 0% for group ≥0 < 30%).

Sensitivity analyses showed that LDL-C lowering in group ≥0 < 30% could not still significantly decrease the volume of CAP with reduction of the CAP volume ranged from −0.010 mm^3^ (SMD, 95% CI: −0.080 ~ 0.061) when the arm of 2007 ILLUSTRATE Ato + Tor was omitted to −0.042 mm^3^ (SMD, 95% CI: −0.108 ~ 0.024) when the arm of 2004 REVERSAL Pro was omitted. No publication bias was found, the values of *p* by Egger’s test for group ≥0 < 30% were 0.537.

Mean levels of LDL-C at baseline and follow up, mean reducing percentage of LDL-C in group <0, ≥0 < 30, ≥30 < 40, ≥40 < 50 and ≥50%, were showed in Table 4.

### The effect of lowering LDL-C by statins on regression of coronary atherosclerotic plaque

**Figure 6:**
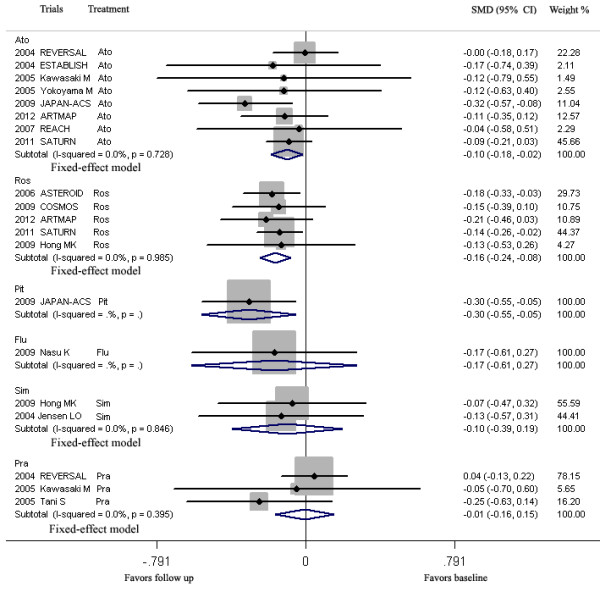
**Meta-analysis of the effects of LDL-C lowering by different statins on the regression of coronary atherosclerotic plaque.** Abbreviations: Ato, Atorvastatin; Ros, Rosuvastatin; Pra, Pravastatin; Pit, Pitavastatin; Sim, Simvastatin; Flu, Fluvastatin; Con, Control; Pac, Pactimibe; Tor, Torcetrapib, Ava 50, 250, 750, Avasimibe 50, 250, 750 mg; Bef, before ACS; Aft, after ACS; Gli, Glimepiride; Pio, Pioglitazone; Rim, Rimonabant.

LDL-C lowering by rosuvastatin, atorvastatin and pitavastatin in group ≤70 and >70 ≤ 100HP mg/dL could lead to regression of CAP, but LDL-C lowering by simvastatin, fluvastatin and pravastatin could not (Figure 6, Table 5).

**Table 5:** Results of meta-analysis in different statins groups

**Group**	**Included arms (and case)**	**Pooled SMD (95% CI, *p *)**	**Heterogeneity test**		**Sensitivity analyses**		**Egger’s test**
			**χ**^***2***^**test ( *p *)**	***I***^***2***^	**Lower SMD (95% CI)**	**Upper SMD (95% CI)**	
Rosuvastatin	5 (1173)	-0.162 (-0.234~ -0.081, 0.000)	0.37 (0.985)	0	-0.153 (-0.249~-0.056)	-0.178 (-0.287~-0.069)	0.770
					Without 2006 ASTEROID Ros	Without 2011 SATURN Ros	
Atorvastatin	8 (1138)	-0.101 (-0.184~ -0.019, 0.016)	4.44 (0.728)	0	-0.075 (-0.162~0.012)	-0.132 (-0.225~-0.038)	0.582
					Without 2009 JAPAN-ACS Ato	Without 2004 REVERSAL Ato	
Pitavastatin	1 (125)	-0.304 (-0.553~-0.055, 0.017)					
Fluvastatin	1 (40)	-0.169 (-0.608~0.270, 0.450)					
Simvastatin	2 (90)	-0.10 (-0.393~ 0.192, 0.501)	0.04 (0.846)	0	-0.074 (-0.467~0.318)	-0.133 (-0.572~0.360)	0.000
					Without 2004 Jensen LO Sim	Without 2009 Hong MK Sim	
Pravastatin	3 (319)	-0.008 (-0.163~0.147, 0.920)	1.86 (0.395)	0	-0.005 (-0.165~0.154)	0.039 (-0.131~0.208)	0.528
					Without 2005 Kawasaki M Pra	Without 2005 Tani S Pra	

LDL-C lowering by rosuvastatin (mean 33.3 mg daily for mean 20 months), atorvastatin (mean 60.3 mg daily for mean 17 months) and pitavastatin (4 mg daily for 8 ~ 12 months) in group ≤70 and >70 ≤ 100HP mg/dL could significantly decrease the volumes of CAP at follow up, compared with the volumes at baseline (SMD −0.162 mm^3^, 95% CI: −0.234 ~ −0.081, *p* = 0.000; SMD −0.101, 95% CI: −0.184 ~ −0.019, *p* = 0.016; SMD −0.304 mm^3^, 95% CI: −0.553 ~ −0.055, *p* = 0.017; respectively). There was no significant heterogeneity among arms (χ^*2*^ for heterogeneity = 0.37, *p* =0.985, *I*^2^ = 0% for rosuvastatin; χ^*2*^ for heterogeneity = 4.44, *p* =0.728, *I*^2^ = 0% for atorvastatin.

Sensitivity analyses suggested that lowering LDL-C by rosuvastatin could lead to regression of CAP with reduction of the plaque volume ranged from −0.153 mm^3^ (SMD, 95% CI: −0.249 ~ −0.056) when the arm of 2006 ASTEROID Ros was omitted to −0.178 mm^3^ (SMD, 95% CI: −0.287 ~ −0.069) when the arm of 2011 SATURN Ros was omitted. Lowering LDL-C by atorvastatin could, but not significantly, lead to regression of CAP when the arm of 2009 JAPAN-ACS Ato was omitted (SMD: −0.075 mm^3^, 95% CI: −0.162 ~ 0.012). No publication bias was found, the values of *p* by Egger’s test for rosuvastatin and atorvastatin group were 0.770, 0.582 respectively (Table 5).

Intensity of lowering LDL-C by different statins was shown in Table 6. Rosuvastatin and atorvastatin could reduce LDL-C by more than 40%.

**Table 6:** Levels and reducing percentage of LDL-C, dosage and duration in different statin group

**Group**	**N**	**Age**	**MeanLDL-C at Baseline (mg)**	**MeanLDL-C at Follow up (mg)**	**Mean Reducing percentage**	**Statin dosage (mg)**	**Duration (month)**
Rosuvastatin	1173	58.1±1.8	123.9±8.6	63.3±7.4	48.4±4.2	33.3±11.6	20.5±6.3
Atorvastatin	1138	58.4±2.5	128.0±14.0	73.0±8.7	42.3±3.7	60.3±28.6	17.5±7.1
Pitavastatin	125	62.5±11.5	130.9±33.3	81.1±23.4	36.2±19.5	4	8~12
Fluvastatin	40	63.0±10.0	144.9±31.5	98.1±12.7	32.3	60	12
Simvastatin	90	57.9±0.1	136.61±5.3	81.2±3.5	39.9±6.1	28.9±10.0	17.8±6.5
Pravastatin	319	58.2±3.2	146.8±7.4	108.9±2.9	24.6±2.6	34.8±9.9	15.4±5.0

## Discussion

### Feature of this meta-analysis

This meta-analysis broke though the limit of single trial, and pooled arms together according to the levels of LDL-C at follow up in the arms, regardless of the measures of lowering LDL-C: treating arm (statins, ACAT inhibitor, CETP inhibitor, decreasing obesity drug, and glucose-lowering agents) and control arms (dietary restriction, moderate LDL-C lowering by statin); intensive and moderate LDL-C lowering. The volumes of CAP at follow up were compared with those at baseline in the same arms to evaluate the regression of the CAPs, this meta-analysis really reflected the change of the plaques volume with the change of LDL-C levels.

Our meta-analysis results indicated that intensive lowering LDL-C in group ≤70, >70 ≤ 100HP mg/dL (mean follow up LDL-C, mean duration of follow up: 60.6 mg/dL, 18.6 months; 77.8 mg/ dL, 17.4 months respectively), ≥30 < 40, ≥40 < 50 and ≥50% (mean LDL-C reducing, mean duration of follow up: 36.1%, 10.3 months; 45.4%, 19.4 months; 53.2%, 24 months respectively) could lead to the regression of CAP; that moderate lowering LDL-C in group >70 ≤ 100MP mg/dL (mean LDL-C reducing by 9.1%, mean 19.8 months of follow up), >100 (mean follow up LDL-C 110.0 and mean 14.6 months of follow up) mg/dL and ≥0 < 30% (mean LDL-C reducing by 10.6%, mean 18.3 months of follow up) could not lead to the regression; and that intensive lowering LDL-C, by mean 48% with rosuvastatin, and by mean 42% with atorvastatin, could regress CAP. The sensitivity analysis confirmed the effect of the LDL-C change on the volume of the plaque.

### The importance of intensive lowing LDL-C on regression of CAP and LDL-C target of this meta-analysis

In the trials that evaluated the effects of LDL-C lowering on atheroma progression by IVUS, the effects varied with level of LDL-C at follow up. In group ≤70 mg, ≥30 < 40% and ≥40 < 50%, the LDL-C at baseline in most trials (including ESTABLISH [14], REVERSAL [13], JAPAN-ACS [12], ASTEROID [37], COSMOS [40], trial by Kawasaki M [16] and by Nasu K [41]) were >120 mg. In ASTEROID [37], COSMOS [40], JAPAN-ACS [12] trial and fluvastatin arm of the trial by Nasu K [41] with respective the mean LDL-C level 60.8 mg, 82.9 mg, 81-84 mg and 98 mg (53.2%, 38.6%, 36% and 32.3% reduction of level of LDL-C) at follow up, it was showed that CAP could be regressed with intensive statin therapy. In ESTABLISH [14] and REVERSAL [13], the mean reducing percent of LDL-C at follow up in the statin treatment arms were 44% and 46% respectively, the volumes of CAPs at follow up were not significantly decreased, compared with those in baseline. In the trails by Yokoyama M [15] and Kawasaki M [16], mean reducing percentage of LDL-C at follow up was 35% for atorvastatin arm of the trial by Yokoyama M [15], 32% for pravastatin arm of the trial by Kawasaki M [16] and 39% for atorvastatin arm of the trial by Kawasaki M [16], the volume of CAPs at follow up were also not significantly decreased, compared with that at baseline. Pooled these arms with follow up LDL-C ≤70 mg or reducing >30% together, these meta-analysis showed that the CAPs could be regressed in group ≤70 mg, ≥30 < 40% and ≥40 < 50%. Because of publication bias in group ≥30 < 40% (Table 3), the level of LDL-C in this group could not be recommended for regressing CAP. Based on the mean level and reducing percentage of LDL-C in group ≤70 mg and ≥40 < 50% (60.6 ± 3.5 mg, 48.8 ± 3.3%; 66.8 ± 8.0 mg, 45.4 ± 2.8%, in Table 4), the meta-analysis in group ≤70 mg and ≥40 < 50% suggested that for regressing CAP, LDL-C should be reduced by >45% or to a target level ≤ 66 mg/dL.

In trials with 18–24 months of non-statin (ACAT inhibitor, decreasing obesity drugs and glucose-lowering agents) treatment, although the levels of LDL-C at follow up in some arms (ACTIVATE [22], STRADIVARIUS [35], PERISCOPE [36], and A-PLUS [21] with daily 50 mg of avasimibe) were >70 ≤ 100 mg/dL, the LDL-C lowering percentage at follow up in the arms were below 30% because the levels of LDL-C at baseline were <95 mg/dL. In ILLUSTRATE trial [23], after treatment with atorvastatin to reduce levels of LDL-C to less than 100 mg/dL, patients were randomly assigned to receive either atorvastatin monotherapy or atorvastatin plus 60 mg of torcetrapib daily. After 24 months, the reduction of LDL-C in both arms was <24% and the progression of CAP was not halted. In trial [34,35] with statins treatment and baseline LDL-C < 110 mg, if the LDL-C lowering percentage at follow up were <24%, the CAP was also not regressed. The meta analysis with six arms in group >70 ≤ 100LP mg/dL and five arms in group >70 ≤ 100MP mg/dL did not show that only >70 ≤ 100 mg/dL of LDL-C level but <30% reduction at follow up could lead to regression of CAP, which further confirmed the importance of intensively lowering LDL-C in regression of CAP. Though LDL-C at follow up in some trials [13,15,16,26,27,39] of LDL-C lowering by statins was >70 ≤ 100 mg/dL and reducing >30%, the CAP in the trials was also not regressed. Included eleven arms with baseline LDL-C >130.0 mg/dL, follow up LDL-C >70 ≤ 100 mg/dL and LDL-C reducing >30% (in group >70 ≤ 100HP mg), this meta-analysis suggested that LDL-C reducing >40% or to target 77.8 mg could regress CAP (Table 4). The meta-analysis in group >70 ≤ 100HP, >70 ≤ 100MP and >70 ≤ 100LP mg/dL indicated that LDL-C reducing percentage, not lowering absolute value of LDL-C at follow up, was important for regressing CAP.

Although rosuvastatin, atorvastatin, pitavastatin, simvastatin, and fluvastatin in some trials could reduce LDL-C level to ≤100 mg or by 30%, the meta-analysis indicated that rosuvastatin, atorvastatin and pitavastatin (mean lowering LDL-C by 48.4%, 42.3% and 36.2% respectively) could regress the CAPs, and simvastatin with mean lowering LDL-C by 39.9% could not. The role of pitavastatin in regressing CAPs remains to be verified because the role was from only one RCT with 125 cases [12]. Pravastatin with mean lowering LDL-C by 24.6% could not regress the CAPs either. Fluvastatin with mean lowering LDL-C by 32.3% in the blinded endpoint trial with 40 patients can regress the CAP [41], but meta-analysis indicated that fluvastatin could not regress the CAP. The reason that pravastatin and fluvastatin in this meta–analysis can not regress the CAPs might be attributed to their low-intensity of lowering LDL-C and low dosage which can not reduce LDL-C by >40%.

Taken all the results of meta-analysis together, it was recommended that LDL-C level should be reduced by >40% or to a target level < 78 mg/dL for regressing CAP.

### The difference of LDL-C target level between this meta-analysis and current guidelines

The patients included in this meta-analysis were coronary heart disease. According to 2004 the guideline of the Adult Treatment Panel III (ATP III) of the National Cholesterol Education Program [43] and 2011 ESC/EAS Guidelines for the management of dyslipidaemias [1], this group of patients belongs to very high risk category, and the recommended targets of LDL-C should be less than 70 mg/dL or 30-40% reduction from baseline in ATP III, and less than 70 mg/dL or a ≥50% reduction in 2011 ESC/EAS Guidelines. The target levels for subjects at very high risk in the both guidelines are extrapolated from several clinical trials [43], mainly from the meta-analysis by Cholesterol Treatment Trialists’ Collaborators [44], which indicated that absolute benefit of LDL-C lowering related chiefly to the absolute reduction of LDL-C, and the risk reductions are proportional to the absolute LDL-C reductions, but the meta-analysis did not provide target level of LDL-C for the benefit in terms of cardiovascular disease reduction [44]. According to 2013 ACC/AHA blood cholesterol guideline [45], this group of patients should be treated with high-intensity statin (atorvastatin 40–80 mg daily or rosuvastatin 20–40 mg daily), which was the intensity of statin suggested in this meta-analysis (Table 6).

The results of our meta-analysis imply that the patients with CHD should be intensively treated with statins (rosuvastatin 33 mg or atorvastatin 60 mg daily) to reduce the level of LDL-C by >40% or to a target level <78 mg/dL for regressing CAP, which have a little different to the guidelines. These different targets level of LDL-C might be due to different observational index: cardiovascular events for both guidelines, CAP volume for this meta-analysis. Moreover, our target is directly from meta-analysis, the target of 2011 ESC/EAS Guidelines is from extrapolation of meta-analysis, not a direct data. Our meta-analysis revealed the relation between the regression of coronary artery disease and LDL-C level from the view of pathological anatomy. Published meta-analysis [17,18] about CAP by IVUS did not review the relationship between LDL-C level and CAP.

### Study limitation

The results of this analysis were obtained by pooling data from twenty clinical trials. As with any meta-analysis, this study has some limitations. Firstly, though no publication bias was observed by Egger’s test there may be a potential of publication bias because only published data were included. Secondly, the methodology used for measurement of coronary atheroma might not be the same in the studies. The plaques volume may be calculated from slices with 1 mm apart for a length of 10 mm vessel in some trials [13,15,22,23,27-29,37], or 0.1-0.3 mm-apart for a length of 10–50 mm vessel in other trials [12,21,33,39,40], which might affect accuracy of plaque measurement. There were some differences in selecting plaque: some trials assessed the plaque in non-culprit vessel, while others assessed non-culprit plaque in a culprit vessel [12,14,34], which assured the plaque was stable. Our study focus on target plaque change, i.e. plaque regression or progression, those differences in measurements and plaque selection did not affect the change of the target plaque with LDL-C levels. So, it has little effect on homogeneous of studies, and this detection bias was very much limited from values of *P* in χ^*2*^ test and *I*^2^ in each group. Thirdly, follow up duration might have some effects of the changes of CAP. Fourthly, other cardiovascular risk factors but LDL-C levels, for example, demographic characteristics such as age, gender and ethnicity, might also affect the effect of LDL-C on CAP, and the effects of these factors on CAP remain to be investigated in future.

### Implication for practice

This meta-analysis investigated the effect of reduction of LDL-C only on the regression of the plaque, not on reduction of cardiovascular events. In fact, all the included trial have no the data about death because only the alive have IVUS data at follow up. But in four-year of the OLIVUS-Ex [46], it was found that patients with annual atheroma progression had more adverse cardio- and cerebrovascular events than the rest of the population. A meta-analysis [47] included 7864 CAD patients showed that rates of plaque volume regression were significantly associated with the incidence of MI or revascularization, and it was concluded that regression of atherosclerotic coronary plaque volume in stable CAD patients may represent a surrogate for myocardial infarction and repeat revascularization. Plaque in CAD, as blood pressure level in hypertension, is not major adverse cardiac events, but does be an important surrogate. Therefore, the conclusion of this meta-analysis not only applies to guide LDL-C lowering therapy for regressing CAP, may also apply to guide LDL-C lowering therapy for reducing major adverse cardio- and cerebrovascular events. Furthermore, high level of LDL-C plays a crucial role in the formation of atherosclerotic plaque, but LDL-C level is not unique risk factor for atherosclerotic plaque. Hypertension is another important risk factor for the formation of plaque [48,49]. Smoking cessation, administrating β-blockers, anti-hypertension therapy might play some role in slowing progression of CAP [48,50-52]. The trend of CAP regression in group <0% might attribute to these non-LDL-C reducing factors.

## Conclusions

Atherosclerotic plaque extension and disruption are basic mechanism of atherosclerotic cardio- and cerebrovascular disease. Stabling and regressing atherosclerotic plaque play an important role in preventing cardio- and cerebrovascular disease. Pooled the twenty trials with CAP detected by gold standard: IVUS, this systemic review demonstrated that intensive lowering LDL-C (rosuvastatin mean 33 mg daily and atorvastatin mean 60 mg daily) with >17 months of duration could lead to the regression of coronary atherosclerotic plaque, LDL-C level should be reduced by >40% or to a target level < 78 mg/dL for regressing CAP.

## Abbreviations

LDL-C: Low-density lipoprotein cholesterol; CAP: Coronary atherosclerotic plaque; CHD: Coronary heart disease; RCT: Randomized controlled trial; CAG: Coronary angiography; IVUS: Intravascular ultrasound; SMD: Standardized mean differences; ACS: Acute coronary syndrome; ACAT: Acyl–coenzyme A:cholesterol acyltransferase; CETP: Cholesteryl ester transfer protein; CI: Confidence interval; ATP III: Adult Treatment Panel III; CAD: Coronary artery disease.

## Competing interests

The authors declare that they have no competing interests. This study was not funded.

## Authors’ contributions

GWQ, FQZ and LYF carried out data extraction, participated in the analysis and drafted the manuscript. LYX and LCY participated in the design of the study and helped to draft the manuscript. HY, CYM and YB conceived the study, and participated in its statistical analysis. All authors read and approved the final manuscript.

## Pre-publication history

The pre-publication history for this paper can be accessed here:

http://www.biomedcentral.com/1471-2261/14/60/prepub
